# Endoplasmic Reticulum-Localized Transmembrane Protein Dpy19L1 Is Required for Neurite Outgrowth

**DOI:** 10.1371/journal.pone.0167985

**Published:** 2016-12-13

**Authors:** Keisuke Watanabe, Norihisa Bizen, Noboru Sato, Hirohide Takebayashi

**Affiliations:** 1 Division of Neurobiology and Anatomy, Graduate School of Medical and Dental Sciences, Niigata University, Niigata, Japan; 2 Division of Gross Anatomy and Morphogenesis, Graduate School of Medical and Dental Sciences, Niigata University, Niigata, Japan; Universidad del Pais Vasco, SPAIN

## Abstract

The endoplasmic reticulum (ER), including the nuclear envelope, is a continuous and intricate membrane-bound organelle responsible for various cellular functions. In neurons, the ER network is found in cell bodies, axons, and dendrites. Recent studies indicate the involvement of the ER network in neuronal development, such as neuronal migration and axonal outgrowth. However, the regulation of neural development by ER-localized proteins is not fully understood. We previously reported that the multi-transmembrane protein Dpy19L1 is required for neuronal migration in the developing mouse cerebral cortex. A Dpy19L family member, Dpy19L2, which is a causative gene for human Globozoospermia, is suggested to act as an anchor of the acrosome to the nuclear envelope. In this study, we found that the patterns of exogenous Dpy19L1 were partially coincident with the ER, including the nuclear envelope in COS-7 cells at the level of the light microscope. The reticular distribution of Dpy19L1 was disrupted by microtubule depolymerization that induces retraction of the ER. Furthermore, Dpy19L1 showed a similar distribution pattern with a ER marker protein in embryonic mouse cortical neurons. Finally, we showed that Dpy19L1 knockdown mediated by siRNA resulted in decreased neurite outgrowth in cultured neurons. These results indicate that transmembrane protein Dpy19L1 is localized to the ER membrane and regulates neurite extension during development.

## Introduction

The endoplasmic reticulum (ER) is a multifunctional organelle responsible for the synthesis of lipids, the modification and trafficking of proteins, and intracellular Ca^2+^ store. ER has a continuous and intricate membrane network, which is broadly subdivided into the following three 3 domains; peripheral cisternae, tubules, and the nuclear envelope [1,2]. The ER network is highly dynamic, constantly changing its morphology, which is highly dependent on microtubules [3,4]. In the nervous system, neurons are highly polarized cells with multiple dendrites and an axon. In neurons, the ER is distributed in axons and dendrites as well as in cell bodies [5–7]. Current studies indicate the involvement of the ER network, including the nuclear envelope in neuronal development, such as neuronal migration and axon growth, which are crucial step for the functional organization of the nervous system [1,8,9]. Microtubule-associated protein P600 tethers microtubules to the ER and regulates neurite extension and migration [10]. In this study, it was observed that knockdown of P600 results in retraction of the ER within neurites and leading processes. The hereditary spastic paraplegia protein, Atlastin-1, which is involved in the formation of the ER network, regulates axonal elongation [11,12]. Furthermore, in neuronal migration during development, the forward movement of the nucleus is the key process, which is referred to as nucleokinesis [13,14]. When nucleokinesis occurs, the microtubule network envelopes the nucleus as a cargo and pulls it forward [15]. In this process, LIS1, dynein, and SUN-Syne complexes mediate coupling between microtubules and the nuclear envelope [16,17]. We previously reported that the putative transmembrane protein Dpy19L1 regulates neuronal migration in the developing mouse cerebral cortex [18]. A Dpy19L family member, Dpy19L2, is an inner nuclear membrane protein in mouse spermatids and is suggested to anchor the acrosomal membrane to the nucleus [19]. These observations raise the possibility that Dpy19L family members may mediate tethering organelles or the cytoskeleton to other membrane-bound organelles. However, the subcellular localization and functions of mammalian Dpy19L1 remain largely unknown.

The multi-transmembrane protein DPY-19 was first identified in *C*. *elegans*. In *dpy-19* mutants, the polarization of Q neuroblasts becomes randomized and results in defective migration, suggesting involvement of *dpy-19* in the polarization and migration of neuroblasts in *C*. *elegans* [20]. The mammalian *Dpy-19-like (Dpy19L)* gene family consists of four members (*Dpy19L1 to Dpy19L4*), and the members have 9−11 putative transmembrane domains [21]. Recently, a *DPY19L2* deletion has been found to cause human globozoospermia, which is a severe male infertility disorder resulting from round-headed spermatozoa [22,23]. In accord with these observations, *Dpy19L2* knockout male mice are sterile caused by aberrant spermiogenesis [19]. Another member of the Dpy19L family, *DPY19L3*, is reported to be associated with human bipolar disorder [24]. More recently, Buettner and colleagues demonstrated that *C*. *elegans* DPY-19 is a novel C-mannosyltranferase, which is able to glycosylate the cell surface receptors MIG-21 and UNC-5 [25,26]. These studies imply the biological importance of the Dpy19L family and molecular functions of mammalian Dpy19L1.

In the present study, we first investigated the subcellular localization of Dpy19L1 in COS-7 cells. Exogenous Dpy19L1 showed a similar pattern with Calreticulin, a marker for the ER, in COS-7 cells. Furthermore, we showed that the subcellular localization of Dpy19L1 was partially coincident with the cytoplasmic ER and nuclear envelope in embryonic mouse cortical neurons. The reticular distribution pattern of Dpy19L1 was disrupted by nocodazole treatment that induces microtubule depolymerization, in both COS-7 cells and cortical neurons. Dpy19L1 knockdown with siRNA inhibited neurite extension in mouse cortical neurons. Our study demonstrates the localization of Dpy19L1 to the ER and suggests its possible significance in neuronal development.

## Results

### Subcellular localization of Dpy19L1 in COS-7 cells

**Fig 1:**
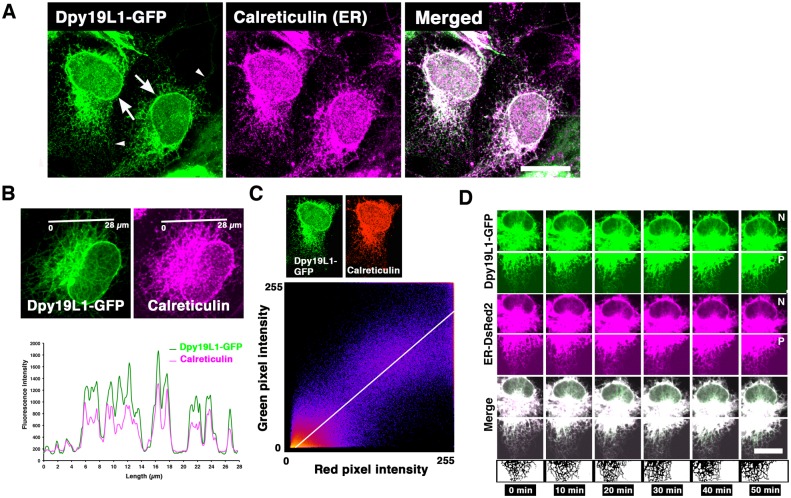
Endoplasmic reticulum (ER) localization of Dpy19L1 in COS-7 cells. COS-7 cells were transfected with a Dpy19L1-GFP plasmid. After 24 h, the subcellular localization of Dpy19L1 was observed by immunofluorescence for GFP and Calreticulin, a marker for the ER. (A) Confocal images of Dpy19L1-GFP (green) and Calreticulin (magenta). Right panel is the merged image. Dpy19L1-GFP shows a similar pattern with Calreticulin. Arrows indicate localization of Dpy19L1 around the nucleus. (B) Double staining of Dpy19L1 and Calreticulin. Lower image indicates intensity profile analysis along a white line in the upper images. (C) Colocalization analysis. An example of scatter plot of red and green pixel intensities of the Dpy19L1-GFP/Calreticulin double-labeled cell shown in Fig 1A. Intensities were measured from fifteen COS-7 cells. (D) Dynamics of Dpy19L1 and the ER were observed by time-lapse imaging. The movies are shown in S1 and S2 Movies. COS-7 cells were transfected with a Dpy19L1-GFP plasmid together with pER-DsRed2. Upper and lower images show perinuclear (N) and peripheral (P) regions, respectively. Time-lapse images were recorded from twenty-five COS-7 cells. Results shown are representative of at least three independent culture experiments. Scale bars: 20 μm in A and C.

**Fig 2:**
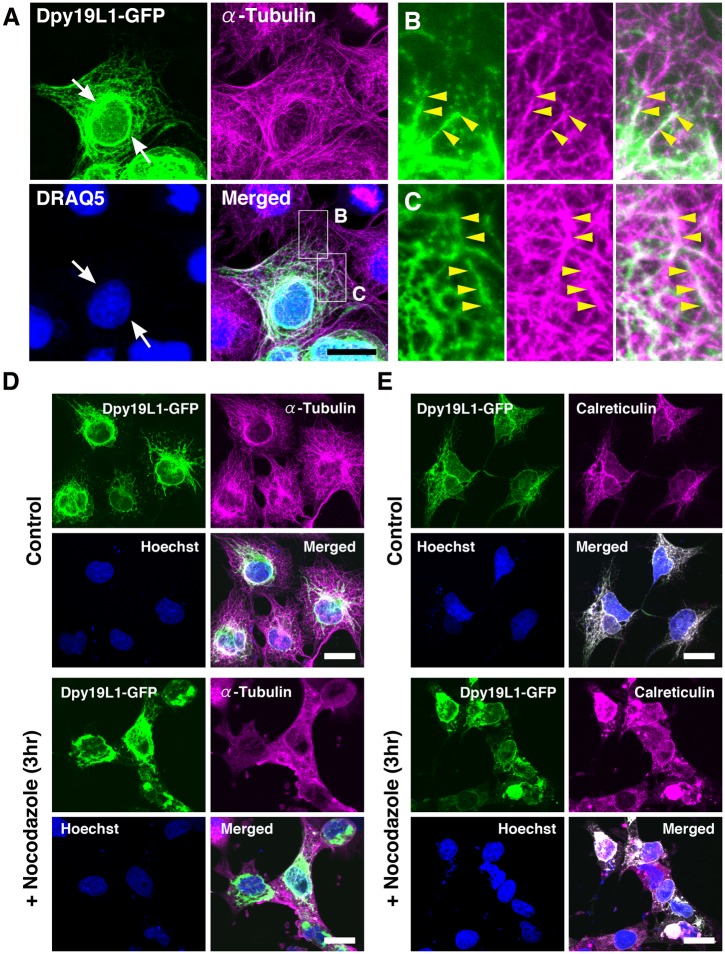
Distribution patterns of Dpy19L1 along microtubules in COS-7 cells. (A-C) Double staining of Dpy19L1-GFP (green) and endogenous α-Tubulin (magenta) in COS-7 cells transfected with Dpy19L1-GFP. Nucleus was labeled by DRAQ5 (blue). Lower right panel shows the merged image. Boxed areas are magnified in B and C. Dpy19L1 is highly localized in a perinuclear region (arrows). The meshwork-like pattern of Dpy19L1 along the microtubule network is observed (yellow arrowheads). (D,E) COS-7 cells transfected with a pDpy19L1-GFP plasmid were treated by nocodazole, an inhibitor of microtubule assembly, for 3 h before fixation. (D) Immunostaining of GFP and α-Tubulin. (E) Immunostaining of GFP and Calreticulin. The cytoplasmic reticular staining of Dpy19L1 is severely disrupted by application of nocodazole. Nucleus was labeled by Hoechst 33342 (blue). Results shown here were obtained from at least three independent culture experiments. Scale bars: 20 μm.

Mammalian Dpy19L1 consists of 746 amino acid residues and has 10 putative transmembrane domains [21]; however, the subcellular localization of this protein is unclear. To reveal where Dpy19L1 is localized, we first examined the subcellular localization of Dpy19L1 protein using confocal microscope. A Dpy19L1-GFP expression plasmid was transfected into COS-7 cells, and 24 h later, the localization was analyzed by immunofluorescence with the anti-GFP antibody. Dpy19L1-GFP fusion protein was found in the perinuclear region and also observed in the cytoplasm in the majority of transfected cells (Fig 1A). Intense GFP signals were observed at the nuclear rims (arrows in Figs 1A and 2A). The distribution in the plasma membrane appeared to be low, whereas it showed a reticular pattern throughout the cytoplasm (Figs 1A and 2A). When observed at high magnification, Dpy19L1 had a punctate pattern in the peripheral regions of the cytoplasm (arrowheads in Fig 1A). Similar results were observed when Dpy19L1, driven by CMV or CAG promoters, was expressed (S1 Fig). Forty-eight h after transfection, some of the transfected cells detached, and Dpy19L1 signal strongly accumulated adjacent to the nucleus (S1 Fig). It is possible that long term and/or high doses of exogenous Dpy19L1 have toxic effects on COS-7 cells. To address a more precise localization, we performed double staining for Dpy19L1 and the ER. Since Dpy19L1-GFP showed the cytoplasmic reticular pattern that is typical for the ER, we assumed that the transmembrane protein Dpy19L1 is localized to the ER, which has a continuous membrane network divided into three domains including the nuclear envelope [1]. Immunofluorescence staining for GFP and Calreticulin, a marker for the ER, showed that a distribution pattern of Dpy19L1-GFP was similar with that of peripheral ER and the nuclear envelope in COS-7 cells (Fig 1A). We confirmed the absence of cross-talk signals between two fluorophores (S2 Fig). Double staining and intensity profile analysis showed that Dpy19L1 was partially colocalized with Calreticulin (Fig 1A and 1B). Furthermore, we drew the scatter plots of red and green pixel intensities from fifteen Dpy19L1-GFP/Calreticulin double-labeled cells. The cluster of points was found along a straight line (Fig 1C; Pearson’s correlation coefficient (R_r_) = 0.77 ± 0.13 (n = 15)), suggesting colocalization of Dpy19L1-GFP and Calreticulin at the level of the light microscope. The clear colocalization was observed at the nuclear rim as well as in peripheral ER, suggesting distribution of Dpy19L1 on the ER membrane including the nuclear envelope. It is known that the ER network is extremely complicated and dynamic [1,4]. We next performed time-lapse imaging to observe Dpy19L1 and the ER directly. COS-7 cells were transfected with both pDpy19L1-GFP and pDsRed2-ER and observed continuously with confocal microscopy. We found that Dpy19L1-GFP also showed dynamic behavior and was coincident with the DsRed2-ER (Fig 1D; S1 and S2 Movies).

The ER network is highly associated with microtubules [4,27]. ER tubules dynamically extend or contract along microtubules, which is essential for structure and functions of the ER. In order to investigate further the subcellular localization of Dpy19L1, we examined whether Dpy19L1 was localized along the microtubule network. COS-7 cells transfected with Dpy19L1-GFP were double stained for α-Tubulin and GFP and observed by confocal microscope. Microtubules labeled with the anti-α-Tubulin antibody were distributed in fine reticular patterns (Fig 2A). Dpy19L1-GFP was observed as reticular or punctate patterns and clearly aligned along microtubules throughout cells (Fig 2A–2C; S3 Movie). Dpy19L1 signals were intensely observed around the nucleus and extended toward the periphery of the cells along microtubules (arrowheads in Fig 2B and 2C). No apparent colocalization of Dpy19L1-GFP and F-actin could be observed in COS-7 cells (S3 Fig). It is reported that treatment with nocodazole, a microtubule-depolymerizing drug, induces retraction of the ER from the periphery of cells toward the nucleus [27]. In COS-7 cells transfected with Dpy19L1-GFP, microtubules were clearly depolymerized following a 3-h treatment with nocodazole before fixation (Fig 2D). Similarly, the cytoplasmic meshwork patterns of both Dpy19L1 and ER (labeled by Calreticulin antibody) were also disrupted by addition of the drug compared with that of the control culture (Fig 2D and 2E). In those cells, Dpy19L1 was predominantly distributed around the nucleus, whereas the cytoplasmic reticular pattern was largely lost. These results, taken together, suggest that Dpy19L1-GFP is preferentially localized to the peripheral ER and the nuclear envelope in COS-7 cells.

### Dpy19L1 localization to the cytoplasmic ER and the nuclear envelope in cortical neurons

**Fig 3:**
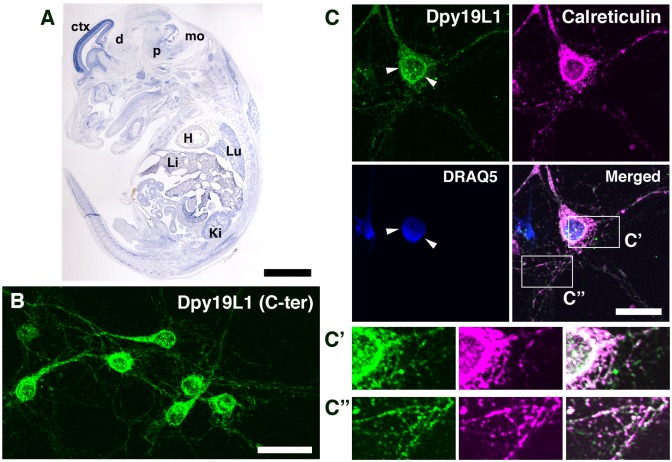
Dpy19L1 distribution in embryonic cortical neurons. (A) Expression of *Dpy19L1* mRNA visualized by ISH on sagittal section of E14.5 mouse embryo. ctx, cerebral cortex; d, diencephalon; p, pons; mo, medulla oblongata; H, heart; Lu, lung; Li, liver; Ki; kidney. (B) Primary neurons were prepared from the E14.5 mouse cerebral cortex and cultured for 5 days. The distribution of Dpy19L1 was observed by immunostaining with anti-Dpy19L1 antibody (C-ter). (C) Double immunostaining for Dpy19L1 (green) and Calreticulin (magenta). Nucleus was labeled by DRAQ5 (blue). Lower right panel shows the merged image. Dpy19L1 is partially colocalized with the ER marker Calreticulin. Arrowheads show Dpy19L1 distribution around the nucleus. C′ and C″ show magnified images of boxed areas. Results shown were obtained from three independent cultures. Scale bars: 200 μm in A, 30 μm in B, and 20 μm in C.

We have previously reported that Dpy19L1 is highly expressed in the developing mouse cerebral cortex and regulates neuronal migration by knockdown experiment [18]. Dpy19L1 was also weakly expressed in peripheral organs, such as lung and kidney, of E14.5 mouse embryos, although the strongest expression was seen in the cerebral cortex at the same stage (Fig 3A). In neurons, the ER network is present in axons and dendrites as well as in cell bodies [5,6], and remodeling of the ER was observed in both axons and dendrites of cultured neurons [28,29]. To study the subcellular localization of Dpy19L1 to the ER in neurons as observed in COS-7 cells, we prepared primary neurons from mouse E14.5 cerebral cortex. We tested two anti-Dpy19L1 antibodies (α-Dpy19L1 (C-ter) and α-Dpy19L1 (N-ter)), which recognize the C-terminal 557−586 and N-terminal 36−85 amino acids of Dpy19L1, respectively. We evaluated the specificity of Dpy19L1 antibodies by both immunostaining and western blotting (S4 Fig). Both α-Dpy19L1 (N-ter) and α-Dpy19L1 (C-ter) showed very similar patterns of staining, and thus α-Dpy19L1 (C-ter) was used for immunocytochemistry in this study (S4 Fig). Dpy19L1 immunoreactivity was intensely localized to the cell bodies of cortical neurons (Fig 3B), in particular, around the nucleus (arrowheads in Fig 3C). In both axons and dendrites, Dpy19L1 showed a punctate distribution similar to that of Dpy19L1 observed in the periphery of COS-7 cells (Fig 3B and 3C). Furthermore, Dpy19L1 and the ER marker Calreticulin were partially colocalized in cell bodies and also in neurites (Fig 3C, 3C′ and 3C″).

**Fig 4:**
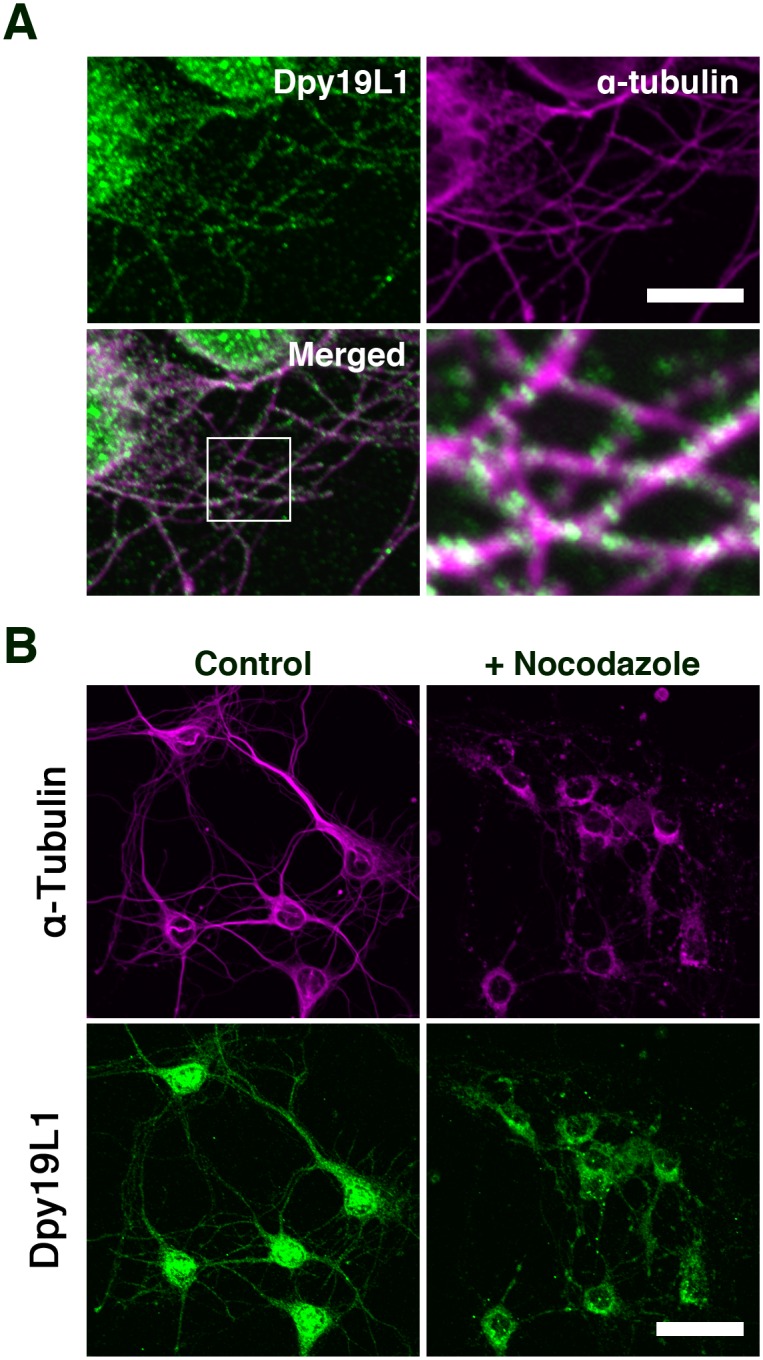
Distribution of Dpy19L1 along microtubules in embryonic cortical neurons. (A) Embryonic cortical neurons were double-labeled with anti-Dpy19L1 (green) and anti-α-Tubulin antibodies (magenta). Lower panel shows the merged image. Boxed area is magnified in lower right image. (B) Primary cortical neurons were treated with nocodazole. The cytoplasmic reticular pattern of Dpy19L1 is perturbed by nocodazole treatment. Results shown were obtained from three independent cultures. Scale bars: 10 μm in A and 30 μm in B.

Microtubules have significant roles in various aspects of neuronal development, including cell migration and axonal outgrowth [30,31]. Microtubule-based nuclear migration, called nucleokinesis, is an essential step for neuronal migration during development [13]. To examine the distribution of Dpy19L1 along the microtubule network as observed in COS-7 cells, we performed double staining of Dpy19L1 and α-Tubulin. Dpy19L1 immunoreactivity showed a punctate pattern, which was clearly along microtubules (Fig 4A). Application of nocodazole led to disrupted localization of Dpy19L1, which was consistent with observations in COS-7 cells (Fig 4B). Nocodazole treatment significantly reduced the reticular distribution of Dpy19L1, whereas control neurons showed the well-organized distribution pattern of both Dpy19L1 and microtubules (Fig 4B). These results suggest that endogenous Dpy19L1 is localized to the cytoplasmic ER and the nuclear envelope in embryonic neurons during development.

### Disruption of neurite extensions by Dpy19L1 downregulation

**Fig 5:**
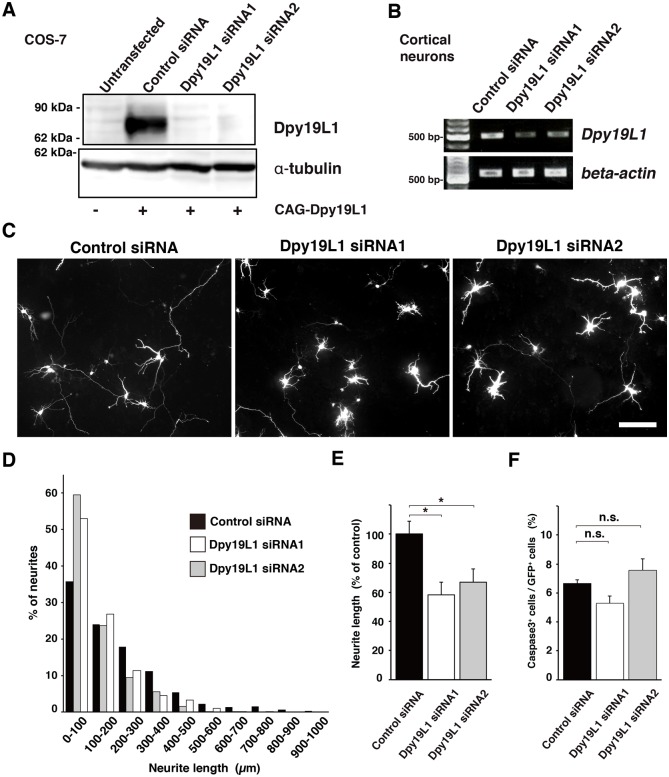
Requirement of Dpy19L1 in neurite extension of cortical neurons. (A) COS-7 cells were transfected with either a Dpy19L1 siRNA or control siRNA along with a CAG-Dpy19L1 expression plasmid, followed by western blot analysis at 48 h after transfection. Dpy19L1 expression was efficiently reduced by Dpy19L1 downregulation. α-Tubulin was used as a control. (B) Either control siRNA or one of two Dpy19L1 siRNAs was transfected in embryonic cortical neurons, and 48 h later, the expression level was checked by RT-PCR. Each Dpy19L1 siRNA decreased *Dpy19L1* expression. β-actin was used as a control. (A, B) The blots (A) and the gels (B) were cropped from the same gels, and the full-length blots and gels are shown in S5B and S5A Fig, respectively. (C−E) E14.5 cortical neurons were transfected with control or Dpy19L1 siRNA and allowed to differentiate for 72 h. The lengths of the longest neurite were compared between control and Dpy19L1-downregulated neurons. Dpy19L1-downregulated neurons show reduced neurite length compared with that of control neurons. Scale bar: 100 μm. (D) Histogram shows distribution of neurite lengths of control and Dpy19L1-downregulated neurons. (E) Average neurite length of control siRNA and Dpy19L1 siRNA transfected neurons. (F) Comparison of the number of cleaved Caspase3-labeled cells between control and Dpy19L1-downregulated neurons at 48 h after transfection. All data shown here are from at least three independent culture experiments. Values are mean ± SEM. *P < 0.05.

To study the physiological function of Dpy19L1 in neural differentiation, we performed siRNA-mediated knockdown of Dpy19L1. First, two siRNAs against Dpy19L1 (Dpy19L1 siRNAs) and a control siRNA as a control were tested in COS-7 cells transfected with Dpy19L1. When each Dpy19L1 siRNA or control siRNA together with a CAG-Dpy19L1 plasmid was transfected in COS-7 cells, Dpy19L1 siRNAs specifically suppressed exogenous Dpy19L1 at the protein level (Dpy19L1 siRNA1: 95.7% ± 2.7% decrease; Dpy19L1 siRNA2: 79.1% ± 7.0% decrease; compared with control siRNA; n = 3), whereas control siRNA did not (Fig 5A).

**Fig 6:**
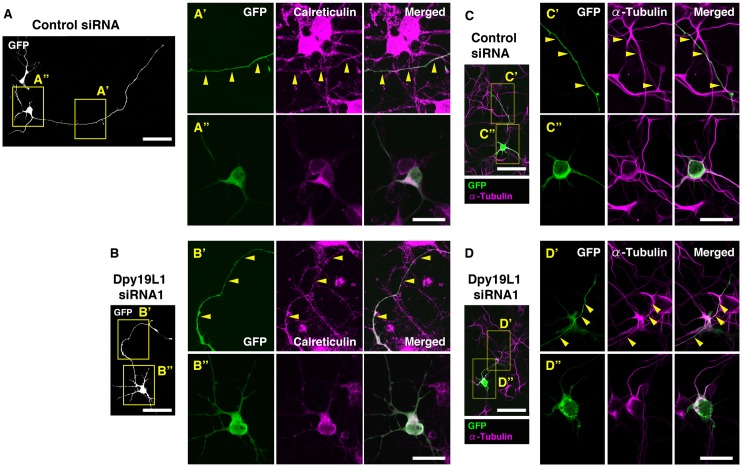
Distribution of Calreticulin in neurites of Dpy19L1-downregulated neurons. Either control siRNA (A and C) or Dpy19L1 siRNA1 (B and D) was transfected in embryonic cortical neurons. Cortical neurons were allowed to differentiate for 72 h, subjected to immunocytochemistry for GFP and Calreticulin (A and B), or for GFP and α-Tubulin (C and D). Boxed areas are magnified in A’-D”. Calreticulin, an ER marker, was observed in neurites of both control and Dpy19L1-downregulated neurons. Results shown are representative of at least three independent culture experiments. Scale bars: 50 μm in A-D, 20 μm in A’-D”.

In *C*. *elegans*, *dpy-19* is involved in polarization of neuroblasts [20]. Furthermore, we previously reported that Dpy19L1 regulates neuronal migration of cortical neurons [18]. These results raise the possibility that Dpy19L1 regulates the morphology of differentiating neurons. To examine this possibility, we next co-transfected either of the Dpy19L1 siRNAs or control siRNA along with pCX-EGFP into embryonic cortical neurons. Forty-eight h later, a significant downregulation of *Dpy19L1* mRNA was observed by treatment of Dpy19L1 siRNA (Fig 5B; Dpy19L1 siRNA1: 46.0% ± 14.6% decrease; Dpy19L1 siRNA2: 54.7% ± 1.4% decrease, compared with control siRNA; n = 3). To estimate neurite outgrowth, the longest neurite, which was presumed to be the axon, was identified by GFP fluorescence. Cortical neurons transfected with both Dpy19L1 siRNA showed a significant reduction in neurite length compared with that of control neurons 72 h post-transfection (Dpy19L1 siRNA1: 41.8% ± 8.71% decrease; Dpy19L1 siRNA2: 33% ± 9.0% decrease, compared with control siRNA; n = 4; Fig 5C–5E). The number of neurons with a long neurite (>400 μm) was decreased in Dpy19L1 downregulated neurons compared with that of control culture (control siRNA: 110/961; Dpy19L1 siRNA1: 20/1025; Dpy19L1 siRNA2: 47/992; Fig 5D). To determine whether knockdown of Dpy19L1 affected the ER network, we observed the distribution pattern of the ER in cortical neurons. Calreticulin was observed in cell bodies and also in neurites of Dpy19L1-downregulated neurons (Dpy19L1 siRNA1: 27/27; Fig 6B) as observed in control neurons (control siRNA: 21/21; Fig 6A), suggesting that Dpy19L1 is not essential for the axonal distribution of the ER. Similarly, we could not find apparent disruptions of microtubule network in neurons transfected with Dpy19L1 siRNA (Fig 6C and 6D). Finally, we determined whether Dpy19L1 downregulation affects cell survival by observing apoptotic cells 48 h after transfection. There was no significant difference between control and Dpy19L1-downregulated neurons in the number of active Caspase-3 positive cells detected immunocytochemically (Fig 5F). These results suggest that Dpy19L1 is involved in neurite outgrowth during embryonic stages.

## Discussion

In this study, we addressed the subcellular localization and physiological function of transmembrane protein Dpy19L1 using mammalian cell lines and cortical neurons. Transmembrane protein Dpy19L1 showed a similar distribution pattern with the ER marker Calreticulin, suggesting localization of Dpy19L1 to the ER including the nuclear envelope both in COS-7 cells and in cultured cortical neurons (Figs 1 and 3). In this study, we observed the patterns of Dpy19L1 in COS-7 cells transfected with a Dpy19L1-GFP plasmid (Figs 1 and 2). Therefore, we cannot exclude the possibility that overexpression of a Dpy19L1-GFP fusion protein led to its subcellular mis-localization in COS-7 cells. However, we observed the reticular distribution patterns of endogenous Dpy19L1 in embryonic cortical neurons (Figs 3 and 4). Based on this observation, a marked mis-localization of Dpy19L1-GFP does not seem to occur, when the localization was analyzed in COS-7 cells at 24 h after transfection. In mouse cultured embryonic cortical neurons, Dpy19L1 was found throughout cells with localization around the nucleus, in the cell body, and neurites (Fig 3). We also observed that Dpy19L1 knockdown attenuated neurite outgrowth of embryonic cortical neurons (Fig 5). However, we could not observe apparent disruptions in the ER network in Dpy19L1-downregulated neurons (Fig 6). Since the efficiency of siRNA-mediated Dpy19L1 knockdown was not so high in our primary culture experiments (Fig 5B), Dpy19L1 protein remaining after Dpy19L1 knockdown may still have functions in neurite outgrowth and/or axonal distribution of the ER. Therefore, complete loss-of-function studies using *Dpy19L1* knockout mice will be required to clarify the physiological roles of Dpy19L1. Our results suggest that the ER-localized protein Dpy19L1 is required for neurite extension during development.

Four Dpy19L members (Dpy19L1-L4) have been identified in both the human and mouse genomes [21]. Among these members, *Dpy19L2* is the most well-studied gene, since *DPY19L2* is a causative gene responsible for human globozoospermia, which is a severe and rare male infertility disorder [22,23,32]. Interestingly, *Dpy19L2* is found only in mammals, whereas three *Dpy19L* members (*Dpy19L1*, *Dpy19L3* and *Dpy19L4*) are in other vertebrates [21]. *Dpy19L2* is generated from an initial duplication of *Dpy19L1* and shares ~72% nucleotide sequence homology with *Dpy19L1* in mouse. These observations led to the assumption that Dpy19L1 and Dpy19L2 may have similarities in their subcellular localization and cellular functions. Dpy19L2 is expressed in mouse sperm, and its localization is restricted to the inner nuclear membrane [19]. *Dpy19L2* knockout male mice are sterile caused by abnormal spermiogenesis, which is in agreement with human globozoospermia. The authors concluded that Dpy19L2 acts as an anchoring protein of the acrosome to the nucleus during spermiogenesis. In this study, we showed that Dpy19L1 was localized to the cytoplasmic ER including the nuclear envelope in mammalian cell lines and mouse cortical neurons (Figs 1, 3 and 5). We also observed the dynamic motion of Dpy19L1-GFP by time-lapse imaging (Fig 1). These results, together with the study of Dpy19L2, suggest that Dpy19L1 mediates the interaction of the ER with other subcellular organelles and/or cytoskeleton. On the other hand, *C*. *elegans* DPY-19 is reported to have C-mannosyltranferase activity [26]. In *C*. *elegans*, *dpy-19* is involved in directed migration of Q neuroblasts [20]. Furthermore, *C*. *elegans* DPY-19 can glycosylate a thrombospondin repeat containing transmembrane protein MIG-21, which is also important for the initial asymmetric migration of Q neuroblasts [20,33]. More recently, human Dpy19L3 was identified as C-mannosyltranferase of R-spondin1, a Wnt signaling regulator [34]. Thus, further studies are needed to elucidate the molecular functions of mammalian Dpy19L1.

The structural organization of the ER is highly dependent on microtubules [24,27]. Several proteins are involved in ER-microtubule interactions; CLIMP-63 is an integral membrane protein and acts as an ER-microtubule linker [35], and REEP1 and Atlastin-1 (hereditary spastic paraplegia proteins) also participate in ER-microtubule interactions [36]. On the other hand, microtubule based nuclear migration, nucleokinesis, is an important process in neuronal migration during development [13]. *LIS1* (The lissencephaly gene) and the motor protein dynein link microtubules to the nucleus and generate the pulling force for nuclear migration [37,38]. RNA interference of *LIS1* inhibits forward nuclear movement of migrating neurons although the leading process continues to grow in brain slice culture [13,38]. Similarly, in *Sun1/2* double knockout mice or *Syne1*^+/-^;*Syne2*^-/-^ mice, defective nucleokinesis has been observed although migrating neurons in *Sun1/2* mutants extended the longer leading processes [17]. These proteins mediate the interaction between the nuclear envelope and microtubule. We previously observed a similar migration defect in some Dpy19L1-downregulated cortical neurons, in which a leading process continued to grow in spite of immobility of a cell body [18]. In this study, we showed localization of Dpy19L1 around the nucleus (Figs 1 and 3). We also showed a punctate distribution pattern of Dpy19L1 along the microtubule network (Figs 2 and 4). Taken together, there is a possibility that the transmembrane protein Dpy19L1 may be involved in the interaction between microtubules and the ER including the nuclear envelope in a direct or indirect manner. However, apparent disruption of ER was not observed in Dpy19L1 knockdown experiments (Fig 6). It will be intriguing to investigate the possible interactions between Dpy19L1 and proteins localized in the nuclear membrane and the microtubule network.

We showed that Dpy19L1 regulates axonal outgrowth in embryonic cortical neurons (Fig 5). The ER is responsible for calcium storage, and calcium-mediated signaling is related to various developmental processes, such as axon growth and guidance [39,40]. In contrast, Atlastin-1, a dynamin-like GTPase, is involved in the formation of the tubular ER network and promotes axonal outgrowth of cultured cortical neurons [2,11]. Microtubule-associated protein P600, which regulates the interaction between the ER and microtubules, is required for neurite extension [10]. It remains an open question as to what mechanisms underlie the defective neurite extension by Dpy19L1 downregulation; we are in the process of identifying these interactions of Dpy19L1. In the present study, we have shown the subcellular distribution and the novel physiological function of Dpy19L1 in neural development.

## Materials and Methods

### Animals

Timed-pregnant ICR mice (Slc:ICR) were obtained from Japan SLC. They were housed on a normal 12 h light/dark schedule with free access to food and water. At noon on the day the vaginal plug was observed was considered embryonic day 0.5 (E0.5). All procedures were performed in accordance with protocols approved by the Animal Research Committee of Niigata University (protocol #27-93-4) and all efforts were made to minimize animal suffering.

### Plasmids

The open reading frame (ORF) of mouse Dpy19L1 (NM_172920) [18] was cloned into the EcoRI and NotI sites of the pCAG-RB (modified pCAG-GS [41]), or the EcoRI and ApaI sites of pEGFP-N1 (TAKARA-Clontech). To obtain efficient expression of Dpy19L1-GFP fusion protein in COS-7 cells, codon-optimized mouse *Dpy19L1* cDNA was synthesized by Genescript and cloned into the above expression vectors. pDsRed2-ER was purchased from TAKARA-Clontech.

### Cell cultures

COS-7 cells were cultured in DMEM (WAKO) containing 10% FBS, Penicillin-Streptomycin (Life Technologies) and maintained at 37°C under 5% CO_2_ [18]. Cells were transfected with pDpy19L1-GFP, pCAG-Dpy19L1, pCX-EGFP, and/or pDsRed2-ER using lipofectamine LTX (Life Technologies) or Fugene 6 (Promega) according to the manufacture’s procedures. After 24−48 h, cells were fixed and then subjected to immunocytochemistry as described below. To inhibit microtubule organization, 5 μg/ml nocodazole (Sigma) was added 3 h for COS-7 cells or 1 h for primary neurons before fixation. An equal amount of dimethyl sulfoxide was added to the control culture.

### Time-lapse imaging

COS-7 cells were plated on a film bottom dish (Matsunami Glass) coated with 15 μg/ml poly-L-ornithine (Sigma). Twenty-four h after transfection, two optical Z-sections at 5-μm intervals were captured at intervals of 5 min for 4 h, using a laser confocal microscope (FV1200, Olympus) equipped with a culture chamber (Tokai Hit), and the images were assembled digitally. Imaging process was performed using Adobe Photoshop CS4.

### Primary culture

Pregnant females were euthanized by an intraperitoneal injection of sodium pentobarbital (125 mg/kg body weight), and the uterine horns were exposed. Embryos were isolated and decapitated in cold 0.01M PBS (pH7.4). The cerebral cortices of E14.5 embryonic mouse brains were dissected and treated with 0.025% trypsin-EDTA (Life Technologies) and 0.01% DNase I (Sigma) for 12–15 min at 37°C. They were dissociated into single cells by gentle trituration and then suspended in Neurobasal medium supplemented with B27 supplements (Life Technologies), Glutamax, and penicillin-streptomycin. They were plated at a density of 25,000 cells/cm^2^ on glass coverslips coated with 15 μg/ml poly-L-ornithine and 1 μg/ml fibronectin (Life Technologies) and cultured for 2−5 days.

### Immunocytochemistry

Cultured cells were fixed with 4% paraformaldehyde (PFA) in 0.01M PBS at room temperature or methanol/acetone (1:1) at −30°C for 15 min, and then rinsed in PBS. For F-actin staining, transfected cells were fixed with 4% PFA, followed by permeabilization with 0.5% TritonX-100 in PBS for 10 min. After blocking with 10% normal goat serum and 0.1% TritonX-100 in PBS, cells were reacted with primary antibodies overnight at 4°C. After washing, cells were labeled with species-specific secondary antibodies conjugated to Alexa Fluor 488 or 594 (1:1000, Life Technologies) and counterstained with Hoechst 33342 (Sigma) or DRAQ5 (Abcam). Primary antibodies used were: rat anti-GFP (1:2000, Nacalai Tesque), rabbit anti-Dpy19L1 (C-ter, 1:100, Abgent; N-ter, 1:250, Abcam), chicken anti-Calreticulin (1:1000, Abcam), mouse anti-Calreticulin (1:200, Abcam), rabbit anti-active Caspase-3 (1:1000, BD Biosciences), Phalloidin (CF dye conjugates, 1:200, Biotium) and mouse anti-α-Tubulin (1:2000, Sigma). Pictures were taken with a digital camera (DP72, Olympus). Confocal images were captured with a confocal laser scanning microscope (FV1200). Intensity profile analysis was performed using FV10-ASW software (Olympus). For colocalization analysis, the scatter plots of red and green pixel intensities of confocal images were created using Image J/Fiji software.

### In situ hybridization (ISH)

Mouse embryos, which were isolated as described above, were fixed with 4% PFA overnight at 4°C, cryoprotected in 20% sucrose, then embedded in OCT compound (Sakura). Frozen sections were cut at 20 μm thickness on a cryostat (CM1850; Leica) and mounted onto MAS-coated glass slides (Matsunami). In situ hybridization for *Dpy19L1* was performed as described previously [18]. DIG-labeled RNA hybrids were reacted with alkaline phosphatase-conjugated anti-DIG antibody (1:2000, Roche). Sections were incubated with nitroblue tetrazolium chloride (NBT; Roche) and 5-bromo-4-chloro-3-indolylphosphate (BCIP; Roche) for color development.

### Western blotting

COS-7 cells were transfected with a pCAG-Dpy19L1 plasmid. Forty-eight h later, they were lyzed with Cell-LyEX MP (TOYO INK). Cell lysates were separated by SDS-PAGE and transferred onto a PVDF membrane (Millipore) by wet transfer method (Bio-Rad). Following blocking with 5% nonfat dry milk, the membrane was incubated with rabbit anti-Dpy19L1 (N-ter, 1:500, Abcam), rabbit anti-Dpy19L1 (C-ter, 1:500, Abgent), or mouse anti-α-Tubulin (1:4000, Sigma) overnight at 4°C and then reacted with the secondary antibody conjugated to horseradish peroxidase (1: 10000, MBL). Signals were developed using Immunostar LD (Wako) and scanned with a C-DiGit Blot Scanner (LI-COR).

### Dpy19L1 siRNA experiments

Synthetic double-stranded small-interfering RNAs (siRNAs: stealth RNAi) against Dpy19L1 were purchased from Life Technologies. The siRNA sequences used were as follows: Dpy19L1 siRNA1 (CCCGGUGCUGUUGUCUUUGCUAUAU) and Dpy19L1 siRNA2 (GGACGUGAAGAAGGAAUUGAUGAAA). A stealth RNAi siRNA negative control (Life Technologies) was used as a control. To assess Dpy19L1-mediated knockdown, COS-7 cells were transfected with 50 nM of either a Dpy19L1 siRNA or a control siRNA along with 4 μg CAG-Dpy19L1 plasmid using lipofectamine 2000. Forty-eight h post-transfection, cells were lyzed with Cell-LyEX MP and subjected to western blot analysis. Data were obtained from three independent cultures.

### Neurite outgrowth assay

Dissociated embryonic cortical neurons were plated at a density of 75,000 cells/cm^2^ on glass coverslips coated with 15 μg/ml poly-L-ornithine. Just after being plated, they were co-transfected with either a Dpy19L1 siRNA or a control siRNA along with pCX-EGFP [20 nM siRNA and 50 ng pCX-EGFP for four-well plate (Thermo Scientific)] using lipofectamine 2000. Six h post-transfection, the transfection medium was replaced with fresh Neurobasal medium supplemented with B27 supplements, followed by incubation for 42 or 66 h (48- or 72-h culture after transfection, respectively) in vitro. Subsequently, neurons were fixed and subjected to immunocytochemistry for GFP and cleaved Caspase-3. Fluorescent images were captured with a digital camera (DP72) or a confocal microscope (FV1200). To evaluate neurite length, the longest neurite from each neuron cultured for 72 h was traced manually and quantified using Image J software. Two hundred to three hundred fifty GFP-positive cortical neurons were scored in each experiment. To estimate cell death in transfected cells, the number of active Caspase-3-positive cells was counted in approximately 280 GFP-positive cells in each experiment. All data shown are the mean ± SEM from at least three independent cultures. Data were statistically analyzed using the Student’s *t* test.

### RT-PCR

Forty-eight h after transfection with the siRNA, total RNA from cultured cortical neurons was extracted with Isogen (Nippon Gene) following the manufacturer’s protocol. Single-stranded cDNAs were made from 2 μg of total RNA using SuperScript Reverse Transcriptase III (Life Technologies) and amplified by PCR. Relative differences of mRNA expression between cells transfected with control and Dpy19L1 siRNA were estimated by semi-quantitative RT-PCR. Primers used for RT-PCR are as follows: *Dpy19L1* (497 bp), 5′-CGGCATTTCTCTCACCTCTC-3′ and 5′-AGAGGTGGCGTCCACATTAC-3′; *beta-actin* (556 bp), 5′-GCCAACCGTGAAAAGATGAC-3′ and 5′-GCACTGTGTTGGCATAGAGG-3′.

## Supporting Information

S1 Fig Distribution of Dpy19L1 in COS-7 cells. (A) COS-7 cells were transfected with a CAG-Dpy19L1 plasmid. After 24 h, the subcellular localization of Dpy19L1 was observed by double staining with anti-Dpy19L1 and anti-α-Tubulin antibodies. A similar distribution pattern is observed with that of Dpy19L1-GFP fusion protein. (B) Forty-eight h post-transfection, Dpy19L1 signal strongly accumulates adjacent to the nucleus (arrows). Results shown here were obtained from four independent cultures. Scale bars: 40 μm in A and 50 μm in B.
(TIF)

S2 Fig Absence of fluorescence cross-talk in immunostaining of GFP and Calreticulin. To check fluorescence cross-talk, we used single-labeled controls. (A,B) Untransfected COS-7 cells were stained with either an anti-GFP (A) or anti-Calreticulin (B) antibody. The fluorescence signals of Calreticulin did not spill over into another channel. (C,D) COS-7 cells transfected with pDpy19L1-GFP were stained with either an anti-GFP (C) or anti-Calreticulin (D) antibody. (C) GFP signals did not show spillover into another channel. Nucleus was labeled by Hoechst 33342 (blue). Scale bars: 50 μm.
(TIF)

S3 Fig Subcellular localization of Dpy19L1 and F-actin in COS-7 cells. Confocal images stained for Dpy19L1-GFP (green) and F-actin (magenta) in COS-7 cells transfected with Dpy19L1-GFP. Apparent colocalization between Dpy19L1 and F-actin is not observed in COS-7 cells. Results shown were obtained from three independent cultures. Scale bar: 20 μm.
(TIF)

S4 Fig Dpy19L1 expression in embryonic cortical neurons. (A) COS-7 cells were transfected with a pCAG-Dpy19L1 plasmid, followed by western blot analysis at 48 h after transfection. Both anti-Dpy19L1 (C-ter) and anti-Dpy19L1 (N-ter) antibodies detected Dpy19L1 protein. α-Tubulin was used as a control. (B) COS-7 cells were transfected with a Dpy19L1-GFP plasmid. After 24 h, double staining with anti-GFP and anti-Dpy19L1 antibodies was performed. Both α-Dpy19L1 (C-ter) and α-Dpy19L1 (N-ter) antibodies detected exogenous Dpy19L1-GFP fusion protein. Both Dpy19L1 antibodies are suitable for immunocytochemistry. (C) E14.5 mouse cortical neurons were immunostained with anti-Dpy19L1 (C-ter; left) or anti-Dpy19L1 (N-ter; right) antibodies. Both α-Dpy19L1 antibodies show similar patterns of staining. (D) A negative control with the omission of incubation with the primary antibody. Nucleus was labeled by Hoechst 33342 (blue). Results shown are representative of at least three independent culture experiments. Scale bars: 30 μm.
(TIF)

S5 Fig Full length images of gels and blots. (A) Fig 5B. Expression of *Dpy19L1* mRNA and *β-actin* mRNA. lane 1: control siRNA, lane 2: Dpy19L1 siRNA1, lane 3: Dpy19L1 siRNA2. (B) Fig 5A. lane 1: untransfected, lane 2: control siRNA + CAG-Dpy19L1, lane 3: Dpy19L1 siRNA1 + CAG-Dpy19L1, lane 4: Dpy19L1 siRNA2 + CAG-Dpy19L1, (lane 5: untransfected).
(TIF)

S1 Movie Time-lapse imaging of Dpy19L1 and the endoplasmic reticulum (ER). COS-7 cells were transfected with both pDpy19L1-GFP and pDsRed2-ER. At 24 h following transfection, both GFP and DsRed2 fluorescences were observed for 4 h.
(MOV)

S2 Movie Time-lapse imaging of Dpy1 9L1 and the endoplasmic reticulum (ER) (Merged). This movie shows merged images presented in Supplemental Movie 2. COS-7 cells were transfected with both pDpy19L1-GFP and pDsRed2-ER. At 24 h following transfection, both GFP and DsRed2 fluorescences were observed for 4 h.
(MOV)

S3 Movie Dpy19L1 localization along the microtubule network. Confocal images of Dpy19L1-GFP (green) and endogenous α-Tubulin (magenta) in COS-7 cells transfected with pDpy19L1-GFP. Nucleus was labeled by DRAQ5 (blue). Eight optical Z-sections were captured at 0.52-μm intervals.
(MOV)
