# Supplementary figures and images for: Endoplasmic Reticulum-Localized Transmembrane Protein Dpy19L1 Is Required for Neurite Outgrowth
Source: PLoS One. 2016 Dec 13;11(12):e0167985. doi: 10.1371/journal.pone.0167985 (PMC5154530; doi:10.1371/journal.pone.0167985)

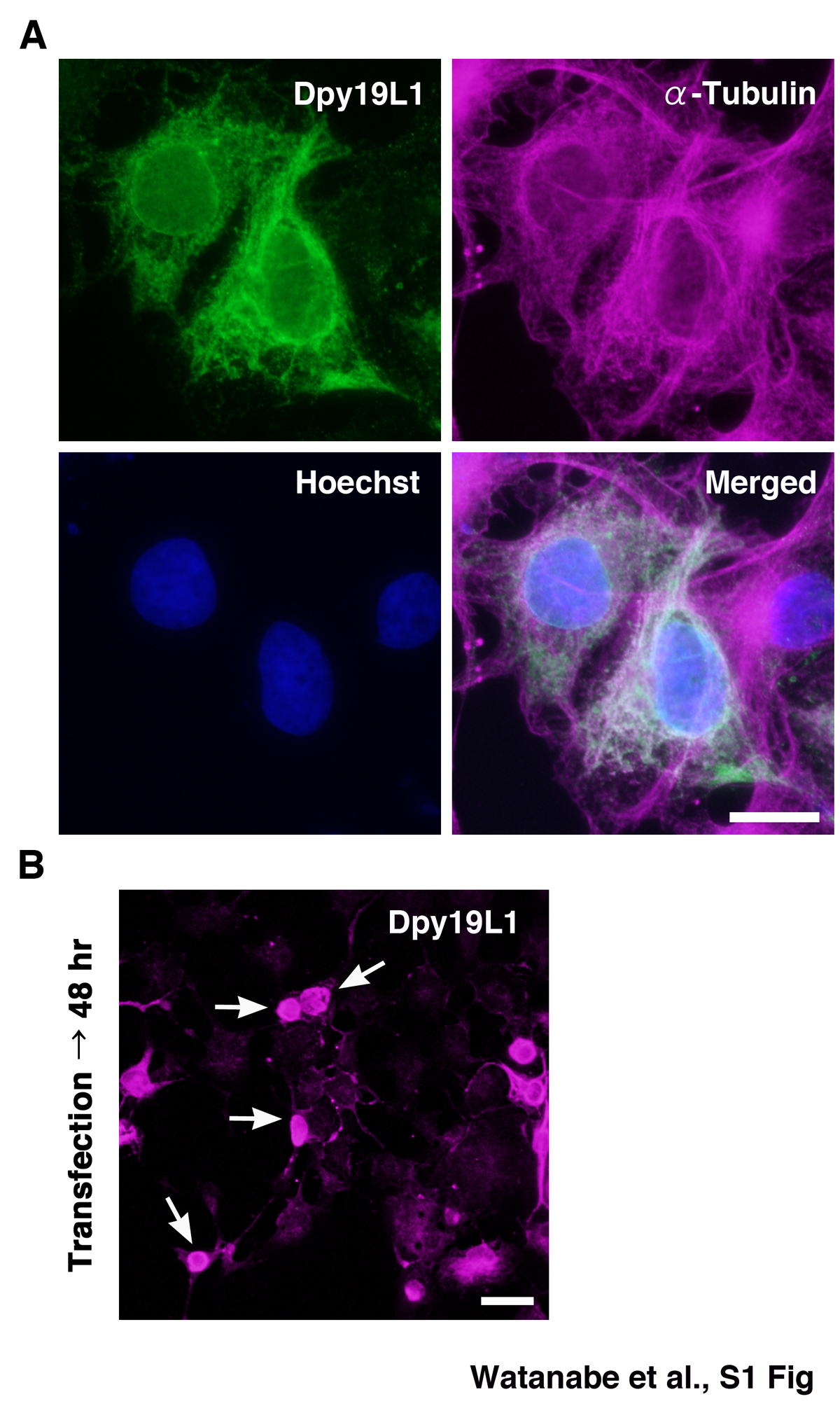

Supplement: S1 Fig — (A) COS-7 cells were transfected with a CAG-Dpy19L1 plasmid. After 24 h, the subcellular localization of Dpy19L1 was observed by double staining with anti-Dpy19L1 and anti-α-Tubulin antibodies. A similar distribution pattern is observed with that of Dpy19L1-GFP fusion protein. (B) Forty-eight h post-transfection, Dpy19L1 signal strongly accumulates adjacent to the nucleus (arrows). Results shown here were obtained from four independent cultures. Scale bars: 40 μm in A and 50 μm in B. (TIF) [file pone.0167985.s001.tif]

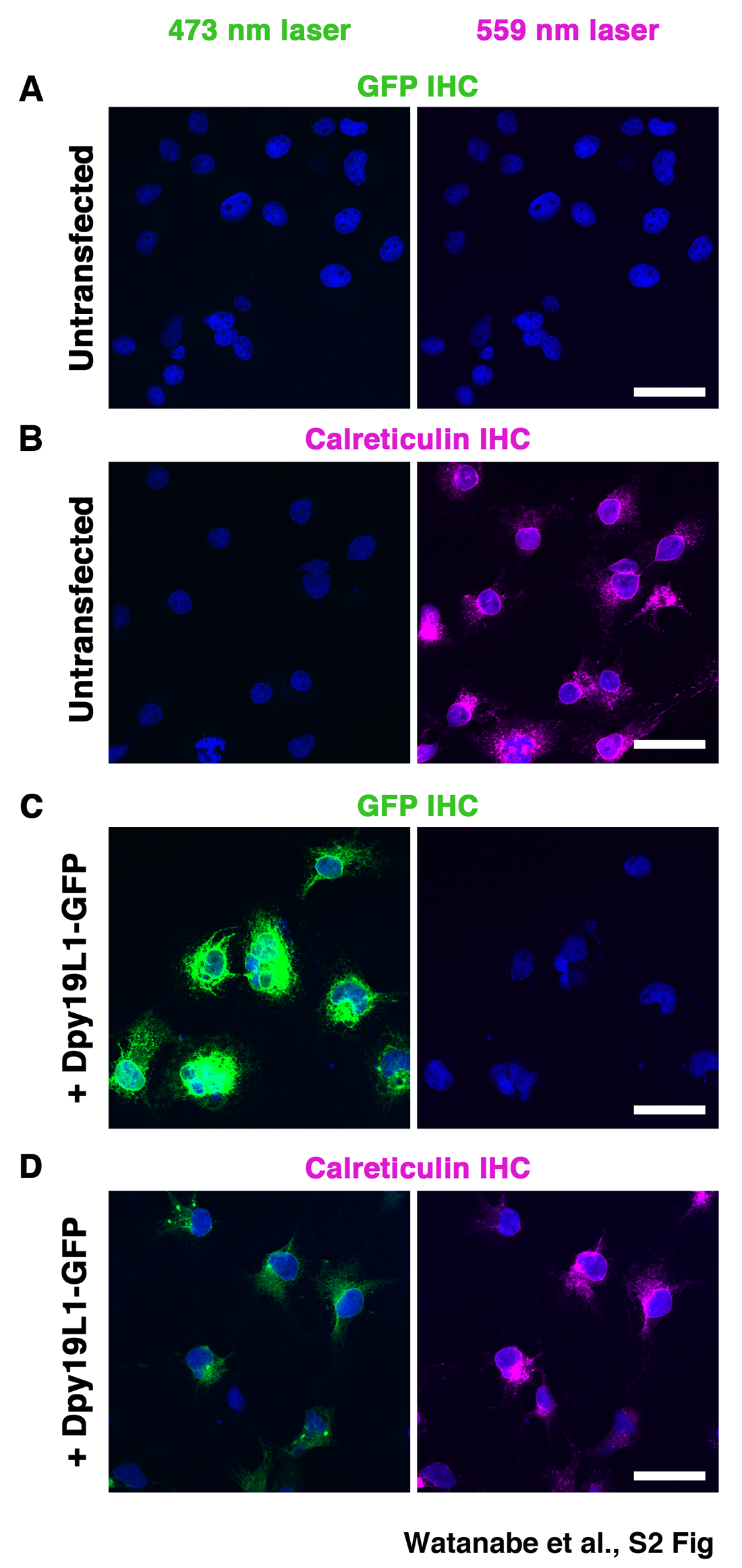

Supplement: S2 Fig — To check fluorescence cross-talk, we used single-labeled controls. (A,B) Untransfected COS-7 cells were stained with either an anti-GFP (A) or anti-Calreticulin (B) antibody. The fluorescence signals of Calreticulin did not spill over into another channel. (C,D) COS-7 cells transfected with pDpy19L1-GFP were stained with either an anti-GFP (C) or anti-Calreticulin (D) antibody. (C) GFP signals did not show spillover into another channel. Nucleus was labeled by Hoechst 33342 (blue). Scale bars: 50 μm. (TIF) [file pone.0167985.s002.tif]

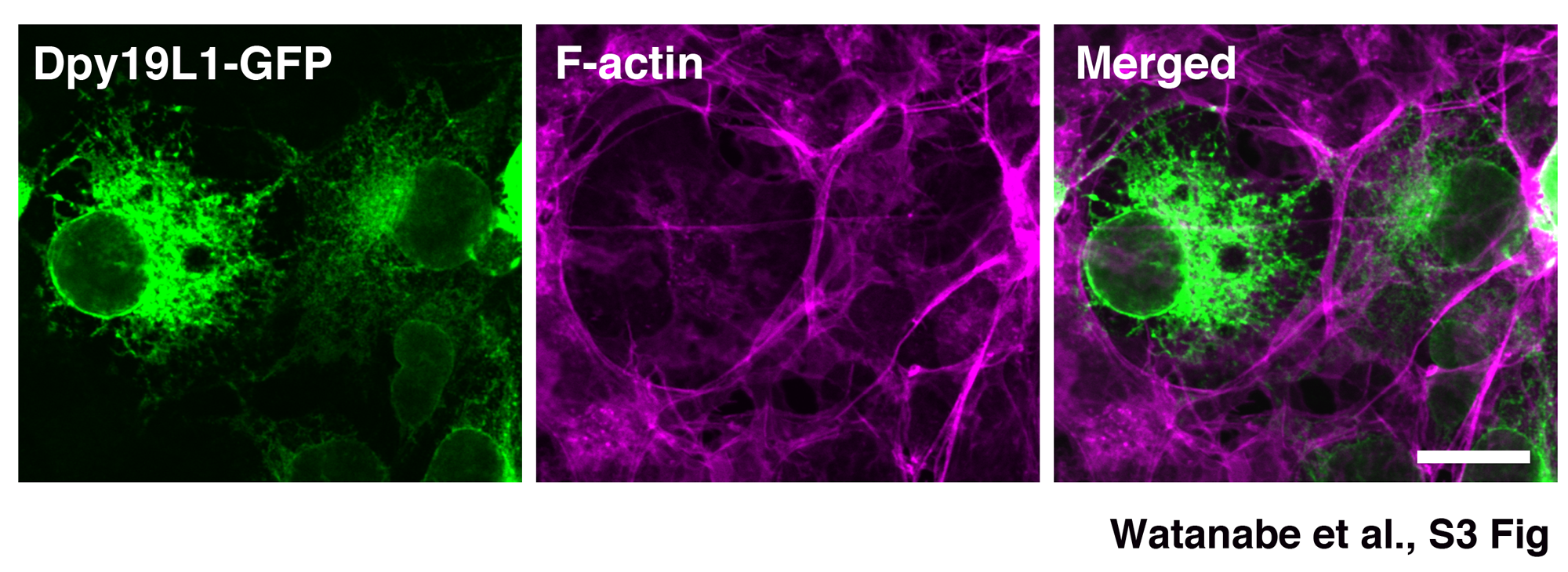

Supplement: S3 Fig — Confocal images stained for Dpy19L1-GFP (green) and F-actin (magenta) in COS-7 cells transfected with Dpy19L1-GFP. Apparent colocalization between Dpy19L1 and F-actin is not observed in COS-7 cells. Results shown were obtained from three independent cultures. Scale bar: 20 μm. (TIF) [file pone.0167985.s003.tif]

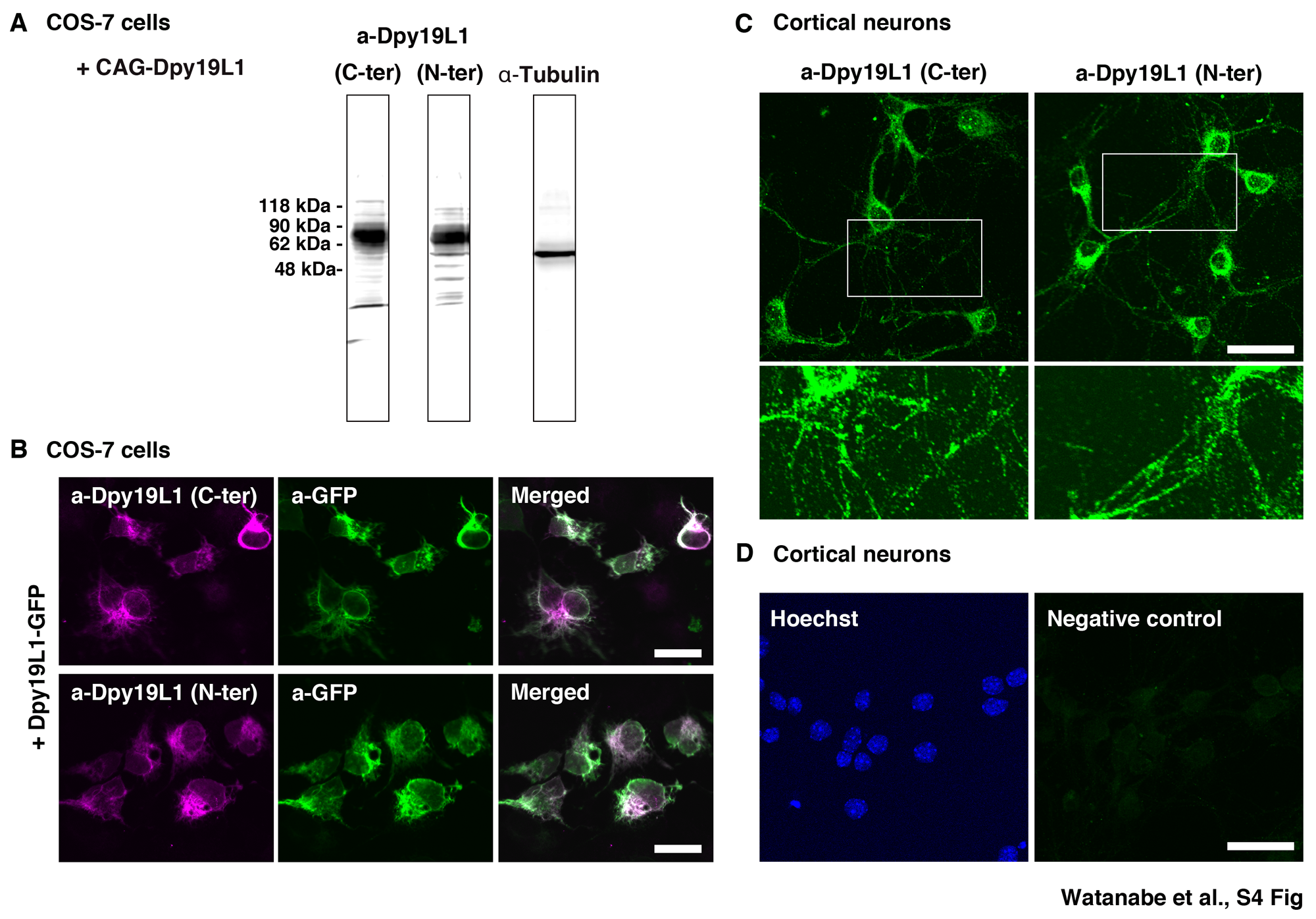

Supplement: S4 Fig — (A) COS-7 cells were transfected with a pCAG-Dpy19L1 plasmid, followed by western blot analysis at 48 h after transfection. Both anti-Dpy19L1 (C-ter) and anti-Dpy19L1 (N-ter) antibodies detected Dpy19L1 protein. α-Tubulin was used as a control. (B) COS-7 cells were transfected with a Dpy19L1-GFP plasmid. After 24 h, double staining with anti-GFP and anti-Dpy19L1 antibodies was performed. Both α-Dpy19L1 (C-ter) and α-Dpy19L1 (N-ter) antibodies detected exogenous Dpy19L1-GFP fusion protein. Both Dpy19L1 antibodies are suitable for immunocytochemistry. (C) E14.5 mouse cortical neurons were immunostained with anti-Dpy19L1 (C-ter; left) or anti-Dpy19L1 (N-ter; right) antibodies. Both α-Dpy19L1 antibodies show similar patterns of staining. (D) A negative control with the omission of incubation with the primary antibody. Nucleus was labeled by Hoechst 33342 (blue). Results shown are representative of at least three independent culture experiments. Scale bars: 30 μm. (TIF) [file pone.0167985.s004.tif]

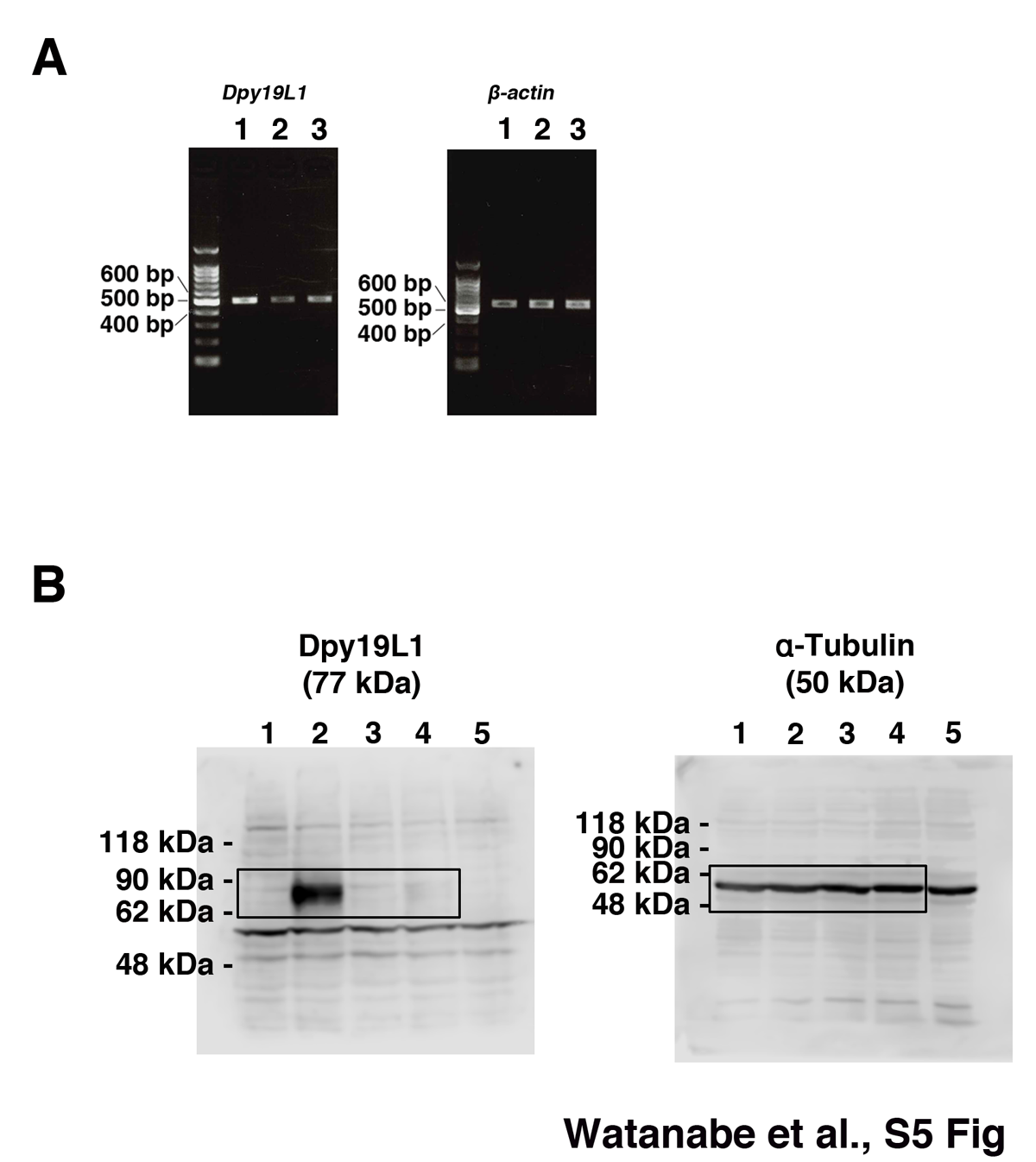

Supplement: S5 Fig — (A) Fig 5B. Expression of Dpy19L1 mRNA and β-actin mRNA. lane 1: control siRNA, lane 2: Dpy19L1 siRNA1, lane 3: Dpy19L1 siRNA2. (B) Fig 5A. lane 1: untransfected, lane 2: control siRNA + CAG-Dpy19L1, lane 3: Dpy19L1 siRNA1 + CAG-Dpy19L1, lane 4: Dpy19L1 siRNA2 + CAG-Dpy19L1, (lane 5: untransfected). (TIF) [file pone.0167985.s005.tif]
